# Nrf2 Inhibitor, Brusatol in Combination with Trastuzumab Exerts Synergistic Antitumor Activity in HER2-Positive Cancers by Inhibiting Nrf2/HO-1 and HER2-AKT/ERK1/2 Pathways

**DOI:** 10.1155/2020/9867595

**Published:** 2020-07-19

**Authors:** Yun Yang, Ziyin Tian, Rui Guo, Feng Ren

**Affiliations:** ^1^School of Basic Medical Sciences, Xinxiang Medical University, Xinxiang, China; ^2^Department of Experimental therapeutics, British Columbia Cancer Research Centre, University of British Columbia, Vancouver, Canada

## Abstract

The HER2-targeting antibody trastuzumab has shown effectiveness in treating HER2-positive breast and gastric cancers; however, its responses are limited. Currently, Nrf2 has been deemed as a key transcription factor in promoting cancer progression and resistance by crosstalk with other proliferative signaling pathways. Brusatol as a novel Nrf2 inhibitor has been deemed as an efficacious and safe drug candidate in cancer therapy. In this study, we firstly reported that brusatol exerted the growth-inhibitory effects on HER2-positive cancer cells by regressing Nrf2/HO-1 and HER2-AKT/ERK1/2 signaling pathways in these cells. More importantly, we found that brusatol synergistically enhanced the antitumor activity of trastuzumab against HER2-positive SK-OV-3 and BT-474 cells, which may be attributed to the inhibition of Nrf2/HO-1 and HER2-AKT/ERK1/2 signaling pathways. Furthermore, the synergistic effects were also observed in BT-474 and SK-OV-3 tumor xenografts. In addition, our results showed that trastuzumab markedly enhanced brusatol-induced ROS accumulation and apoptosis level, which could further explain the synergistic effects. To conclude, the study provided a new insight on exploring Nrf2 inhibition in combination with HER2-targeted trastuzumab as a potential clinical treatment regimen in treating HER2-positive cancers.

## 1. Introduction

HER2 is an important member of the ErbB family implicated in several types of human cancer such as breast cancer, gastric cancer, and ovarian cancer [1–3]. Homodimers or heterodimers with other ErbB receptors results in phosphorylation of residues in the intracellular domain and then recruitment of adapter molecules responsible for activation of PI3K/AKT pathway or ERK1/2 signaling pathways involved in cancer cell proliferation and survival [4–6]. Trastuzumab, a recombinant HER2-targeted humanized antibody, was able to bind to the extracellular domain IV of HER2 and then result in inhibition of downstream AKT or ERK1/2 signaling [7, 8]. It has been approved by the US Food and Drug Administration (FDA) for clinical use for patients with HER2-overexpressing metastatic breast cancer (MBC) in 1998. Despite the considerable clinical efficacy, the majority of patients who have an initial response to trastuzumab therapy developed resistance within one year of treatment [9, 10]. Hellstrom et al. revealed that ovarian cancer cell lines from stage III and IV patients with HER2 overexpression were sensitive to trastuzumab by antibody-dependent cellular cytotoxicity (ADCC) *in vitro* [11]. Although the modest therapeutic effects of trastuzumab on ovarian cancer cells were observed in preclinical studies, the existing data in clinical trials point to more effective HER2-directed drug or regimen [12]. Collectively, there is an urgent need to enhance the efficacy of trastuzumab in treating HER2-amplified cancers.

Nuclear factor erythroid 2-related factor 2 (Nrf2) was a redox-sensitive master regulator of a variety of crucial antioxidant molecules. Kelch-like ECH-associated protein 1 (Keap1) as a cysteine-rich repressor protein binds to Nrf2. Under normal conditions, Keap1 retains Nrf2 in the cytoplasm and promotes ubiquitination and eventual degradation of Nrf2 [13, 14]. However, under the stressful condition, the steady-state level was disrupted and Nrf2 is released from Keap1 and transferred to the nucleus where it binds to conserved ARE sequences [14–16]. In the nucleus, Nrf2 upregulates target gene expression by binding to the antioxidant response element (ARE) of a series of antioxidant enzymes, such as NAD(P)H: quinine oxidoreductase-1 (NQO1), glutathione S-transferase (GST), and hemeoxygenase-1 (HO-1) [17]. Enhanced expression of HO-1 contributes to the survival of cancer cells and inhibits apoptosis [18]. On one hand, Nrf2 transactivates a variety of antioxidant genes involved in defensive and adaptive pathways in response to oxidative stress in normal cells [19]. On the other hand, Nrf2 was always deemed as an activator in cancer progression, which promotes the aberrant proliferation and metastasis of cancer cells [20, 21]. Notably, recent studies also indicated that Nrf2 inhibitors enhance the sensitivity of cancer cells to chemotherapeutic drugs [22, 23]. Hou et al. reported that PMF, as a Nrf2 inhibitor, could be used as an effective adjuvant sensitizer to enhance the effects of cisplatin in lung cancer A549 cells and promotes apoptosis eventually [24]. Arlt et al. also revealed that inhibition of Nrf2 by the alkaloid trigonelline renders pancreatic cancer cells more susceptible to apoptosis [25]. Manandhar et al. revealed that Nrf2 inhibition represses HER2 signaling in ovarian carcinoma cells, suggesting that Nrf2 modulation might be a therapeutic strategy to limit tumor growth in ovarian cancers [26]. Bao et al. reported that the novel Nrf2 target gene, ABCF2, plays a critical role in cisplatin resistance in ovarian cancer, and targeting Nrf2 signaling may be a potential strategy to improve chemotherapeutic efficiency in ovarian cancer [27]. Su et al. revealed that Nrf2 suppressor reversed chemoresistance in CDDP-resistant cervical cancer cells by inactivating PI3K/AKT pathway [28]. Therefore, these results above suggest that the inhibition of Nrf2 may enhance the efficacy of chemotherapeutic drugs or renders cancer cells susceptible to apoptosis.

Brusatol was a quassinoid plant extract from Brucea javanica that was usually used in Traditional Chinese Medicine for treating amoebic dysentery, cancer, and malaria [29, 30]. Recently, brusatol was found to reduce the Nrf2 protein level by enhancing ubiquitination and degradation of Nrf2 in a Keap1-independent way [31, 32]. Wu et al. revealed that brusatol has the capacity to decrease the Nrf2 expression level and enhanced the cytotoxicity of Taxol [33]. Xiang et al. showed that brusatol effectively enhances the anticancer effects of gemcitabine through inhibiting gemcitabine-induced Nrf2 activation in pancreatic cancers [34]. Also, brusatol shows the potency on enhancing the toxicity of irinotecan and inducing cell death in human colon cancer cells [35]. Collectively, these results suggest that brusatol may have the potential to be developed into an adjuvant chemotherapeutic drug against cancer.

Previously, our study revealed that synergistic antitumor activity of trastuzumab plus nimotuzumab may be attributed to the inhibition of the crosstalk of HER2-ERK1/2 signaling pathway and Nrf2-dependent antioxidant responses pathway [2]. In this study, we are the first to investigate the effects of Nrf2 inhibition by brusatol in HER2-positive cancers. Results revealing that brusatol was effective in inhibiting HER2-positive breast cancer BT-474 and SK-BR-3 cells and ovarian cancer SK-OV-3 cells. Especially, we also found that HER2-AKT/ERK1/2 signaling was inhibited, which suggested a new mechanism of brusatol. As we know, trastuzumab targeted the extracellular domain of HER2 and inhibited the activation of HER2-AKT/ERK1/2 signaling pathway. Therefore, we seek to examine if trastuzumab in combination with brusatol may exert the synergistic effects on these HER2-positive cancers. Results revealed that brusatol synergistically enhanced the growth-inhibitory effect of trastuzumab against BT-474 and SK-OV-3 cancer cells *in vitro* and *in vivo*. We observed a significant decrease on the phosphorylation of AKT and ERK1/2 in BT-474 and SK-OV-3 cells when treated with trastuzumab plus brusatol compared to either agent alone. Nrf2/HO-1 signaling was also inhibited more effectively when treated with trastuzumab plus brusatol that further resulted in significant ROS accumulation and apoptosis. Furthermore, we found that knockdown of Nrf2 by siRNA enhanced the effects of trastuzumab which also revealed that brusatol may exert the synergistic effects with trastuzumab by targeting Nrf2. In summary, these results above suggested that the therapeutic strategy by combining trastuzumab with brusatol has a great potential to treat HER2-positive cancers in clinics.

## 2. Materials and Methods

### 2.1. Cell Lines

The human breast cancer cell line BT-474 and SK-BR-3 and ovarian cancer cell line SK-OV-3 were purchased from the American Type Culture Collection (ATCC). Cells were cultured with RPMI-1640 or DMEM medium (Gibco; Thermo Fisher Scientific, Inc.) supplemented with 10% Fetal Bovine Serum (Gibco; Thermo Fisher Scientific, Inc.) in an incubator at 37°C with 8% CO_2_.

### 2.2. Agents

Trastuzumab was purchased from Roche (Basel, Switzerland). Brusatol was purchased from Yuanye Biotech Corporation (Shanghai, China). It is over 95% pure determined by HPLC. The stock solution of brusatol was prepared by dissolving in DMEM with 0.25% dimethyl sulfoxide (DMSO). All primary antibodies were purchased from Cell Signaling Technology (Beverly, MA, USA) including the antibodies against Nrf2 (1 : 1,000; cat. no. 12721), HO-1 (1 : 1,000; cat. no. 43966), HER2 (1 : 1,000; cat. no. 2242), phosphorylated (p)-HER2 (1 : 1,000; cat. no. 2243), AKT (1 : 1,000; cat. no. 9272), p-AKT (1 : 1,000; cat. no. 4060), ERK1/2 (1 : 1,000; cat. no. 4695), p-ERK1/2 (1 : 1,000; cat. no. 8544), and *β*-actin (1 : 2,000; cat. no. 3700). Horseradish peroxidase-conjugated goat antimouse/rabbit secondary antibodies were purchased from ProteinTech (1 : 5,000; cat. no. SA00001-1 or SA00001-2). SiRNA was ordered from RiboBio (Guangzhou, China).

### 2.3. Animals

Six-week-old female BALB/c nude mice were obtained from the Beijing Vital River Laboratory Animal Technology (Beijing, China). The animal research was conducted according to the Principle of Laboratory Animal Care (NIH Publication No. 85-23, revised in 1985). All experimental protocols were approved by the Animal Experimentation Ethics Committee of Xinxiang Medical University and all efforts were made to minimize animal suffering and reduce the number of animals used. Animals were treated in accordance with the guideline of the Animal Care and Use Committee of Xinxiang Medical University.

### 2.4. *In Vitro* Cytotoxicity Assay

Breast cancer SK-BR-3 and BT-474 cells and ovarian cancer SK-OV-3 cells were plated in 96-well plates (5 × 10^3^ cells per well) and incubated with trastuzumab, brusatol, or trastuzumab in combination with brusatol for 48 h. Cell viability was then determined by CCK-8 kit (Dojindo). The percentage of surviving cells was calculated using the following formula: [(A450 of experiment–A450 of background)/(A450 of control–A450 of background)] × 100. Combination index (CI) values were calculated using the Chou-Talalay method by Compusyn Software. Drug synergy, addition, and antagonism are defined by CI values less than 1.0, equal to 1.0, or greater than 1.0, respectively.

### 2.5. Transfection with Small Interfering RNA

The target small interfering RNA (siRNA) sequences directed against human Nrf2 were 5′-GAGAAAGAATTGCCTGTAA-3′ and 5′-TCCCGTTTGTAGATGACAA-3′. A scramble siRNA was purchased from RiboBio (Guangzhou, China) as control. Cells were transfected using Lipofectamine 2000 (Invitrogen, USA) according to the manufacturer's instructions. The final concentration of the siRNAs was 20 nmol/L.

### 2.6. Immunoblotting

Firstly, cells were lysed in RIPA lysis buffer containing 50 mmol/L Tris-HCl pH 7.4, 150 mmol/L NaCl, 0.1% SDS, 1% NP-40, 0.5% deoxycholic acid sodium salt (*w*/*v*)] supplemented with 2 *μ*L/mL protease inhibitor cocktail (Sigma) and 10 *μ*L/mL phosphatase inhibitor cocktail (Sigma). The protein concentration of cell lysates was measured by a QuantiPro BCA protein assay kit (Sigma Aldrich). After denaturation, total cell lysates were subjected to SDS–polyacrylamide and immunoblotted with primary antibodies and HRP-conjugated secondary antibody. After washing of the membrane, the bands were detected using the sensitive ECL reagent (Millipore) and visualized using an ChemiDoc imaging system (Bio-Rad Laboratories, Inc.).

### 2.7. Reactive Oxygen Species (ROS) Detection

The production of ROS in solution was routinely detected with 2′, 7′-dichlorodihydrofluorescein diacetate (DCFH-DA) (Sigma Aldrich) according to the instruction of supplier. Briefly, the cells (1 × 10^5^/well) were treated with control IgG, brusatol, trastuzumab, or brusatol plus trastuzumab at 37°C for 24 hours. Then, 1 mM DCFH-DA was added to the cells (final concentration of DCFH-DA will be 10 *μ*M). After incubation for 30 min, the cells were washed with phosphate buffer saline (PBS) and collected. Fluorescence signal intensities indicating ROS levels were recorded by flow cytometer (BD Biosciences) using excitation and emission spectra of 488/525 nm.

### 2.8. Apoptosis Analysis

Cells were seeded in 6-well plates at a density of 3 × 10^5^/well and exposed to different agents. After incubation for 48 hours, the cells were washed with ice-cold PBS and incubated with annexin V and propidium iodide (PI) (Sigma Aldrich) for 15 min at room temperature in the dark. The rate of apoptotic cells was determined by flow cytometer (BD Biosciences) and analyzed by FlowJo software. The percentage of early apoptosis was calculated by annexin V (+)/PI (−), while the percentage of late apoptosis was calculated by annexin V (+)/PI (+).

### 2.9. *In Vivo* Therapy Study

BT-474 or SK-OV-3 cells (5 × 10^7^ per mouse) were inoculated subcutaneously into the right flank of female BALB/c nude mice. When tumor volumes reached an average of about 120 mm^3^ approximately on day 10 after inoculation, the mice were randomly divided into four groups of five mice each. Mice were intraperitoneally injected with control IgG (15 mg/kg for three times every week), trastuzumab (15 mg/kg for three times every week), brusatol (2 mg/kg once daily), or the combination of trastuzumab (15 mg/kg for three times every week) with brusatol (2 mg/kg once daily) for 1 week. Tumors were measured with digital calipers, and tumor volumes were calculated by the formula: volume = [length × (width)^2^]/2. Mice were euthanized with CO_2_ asphyxiation.

### 2.10. Statistical Analysis

Statistical analysis was performed with the SPSS 20.0 software (SPSS) or Graphpad Prism version 5.0 (Graphpad software). Data are shown as mean ± SD. One-way or two-way ANOVA and Student's *t*-test were used to analyze differences between two experimental groups. Differences were considered significant at *p* < 0.05 (∗). Nonlinear regression analyses were used to fit curves.

## 3. Results

### 3.1. Brusatol Exhibits Effective Growth-Inhibitory Activity against HER2-Positive Cancer Cells through Inhibiting Nrf2/HO-1 Antioxidant Pathway and HER2/AKT/ERK1/2 Signaling Pathway

**Figure 1:**
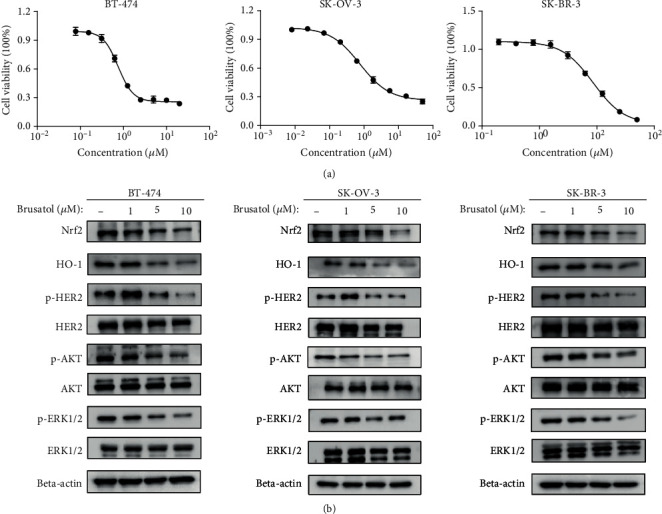
Brusatol regressed the growth of HER2-positive SK-OV-3, BT-474, and SK-BR-3 cells by inhibiting Nrf2/HO-1 and HER2-AKT/ERK1/2 pathways. (a) BT-474, SK-OV-3, and SK-BR-3 cells were treated with brusatol in a dose range for 2 days. IC50 for BT-474, SK-OV-3, and SK-BR-3 were 0.7537 *μ*M (95% confidence interval [CI], 0.6983–0.8136 *μ*M), 0.7610 *μ*M (95% CI, 0.6699–0.8646 *μ*M), 8.631 *μ*M (95% CI, 7.699–9.675 *μ*M), respectively. Points, mean of 3 independent CCK-8 assays; bars, SD. (b) BT-474, SK-OV-3, and SK-BR-3 cells were treated with brusatol at 0, 1, 5, or 10 *μ*M for 24 hours. The changes in Nrf2/HO-1 and HER2-AKT/ERK1/2 signaling pathways were monitored by western blotting.

To investigate the antitumor effects of brusatol against HER2-positive cancer cells *in vitro*, we utilized CCK-8 assay to evaluate the activity of brusatol on cell viability and growth. Results showed that brusatol exhibited potent inhibitory effects in a dose-dependent manner either in BT-474, SK-OV-3, or SK-BR-3 cell line which overexpressed HER2 (Figure 1(a)).

Furthermore, we also observed that brusatol treatment caused a marked downregulation of Nrf2 level and regressed the expression of HO-1, which is a Nrf2-regulated major protein in cells. Previously, our studies suggested the potential crosstalk of Nrf2 antioxidant pathway with HER2/ERK1/2 signaling pathway may exist in esophageal and breast cancer cells [2, 15]. Interestingly, in the study, we also found that brusatol may exert its antitumor effects through inhibiting phosphorylation of HER2 and HER2 downstream pathways, as indicated by the regression of phosphorylated AKT and ERK1/2 (Figure 1(b)). Collectively, these results suggested a possible mechanism of brusatol on HER2-positive cancer cells, which functions by repressing HER2-AKT/ERK1/2 signaling pathway.

### 3.2. Trastuzumab and Brusatol Act Synergistically on BT-474 and SK-OV-3 Cancer Cells *In Vitro*

**Figure 2:**
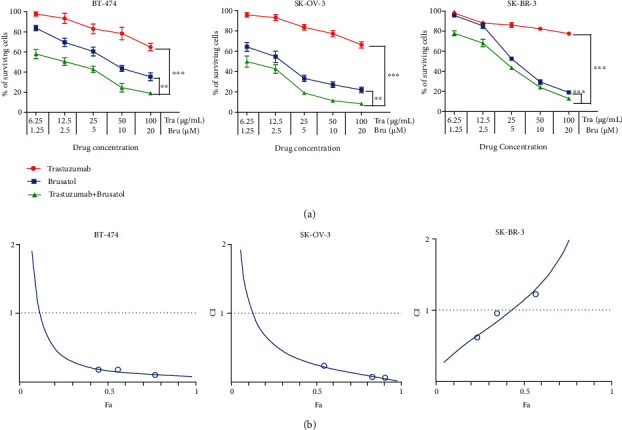
Brusatol exhibited antitumor activity in combination with trastuzumab in a synergistic manner on HER2-positive BT-474 and SK-OV-3 cells. (a) BT-474, SK-OV-3, and SK-BR-3 cells were treated with trastuzumab and brusatol as single agents and in combination with trastuzumab in a dose range for 2 days. Points, mean of 3 independent CCK-8 assays; bars, SD. ∗*p* < 0.05, ∗∗*p* < 0.01, ∗∗∗*p* < 0.001. (b) The synergistic effect of trastuzumab in combination with brusatol was evaluated on the growth of BT474, SK-OV-3, or SK-BR-3 cell line. Combination index (CI) values were calculated at the drug concentration of trastuzumab (12.5 *μ*g/mL) plus brusatol (2.5 *μ*M), trastuzumab (25 *μ*g/mL) plus brusatol (5 *μ*M), and trastuzumab (50 *μ*g/mL) plus brusatol (10 *μ*M) using the Chou-Talalay method. Drug synergy, addition, and antagonism are defined by CI values less than 1.0, equal to 1.0, or greater than 1.0, respectively.

Although trastuzumab has shown considerable clinical efficacy in HER2-overexpressing breast cancers, the overall response rate is still limited in many breast cancer patients [36]. According to the results above, brusatol has the potency on inhibiting the activation of HER2 and HER2 downstream pathway, which was similar to that of trastuzumab. Therefore, we speculated that brusatol may have the potential to enhance trastuzumab efficacy in treating HER2-positive cancers. Results showed that the inhibitory effect of trastuzumab in combination with brusatol was significantly greater than that of either agent alone in both SK-OV-3 and BT-474 cancer cells, while the combinatorial treatment hardly shows superior activity compared to brusatol on SK-BR-3 cells (Figure 2(a)). The combination index (CI) is a mathematical method commonly used to measure the pharmacological interaction of two drugs [9, 37]. As shown in Figure 2(b), the method of Chou and Talalay analysis showed that the CI for every combination treatment in BT-474 and SK-OV-3 cells was <1, indicating significant synergistic effects of these combination treatments. Collectively, it is clear from the results that the superior effects of trastuzumab plus brusatol were synergistic on BT-474 and SK-OV-3 cells (Figure 2(b)).

**Figure 3:**
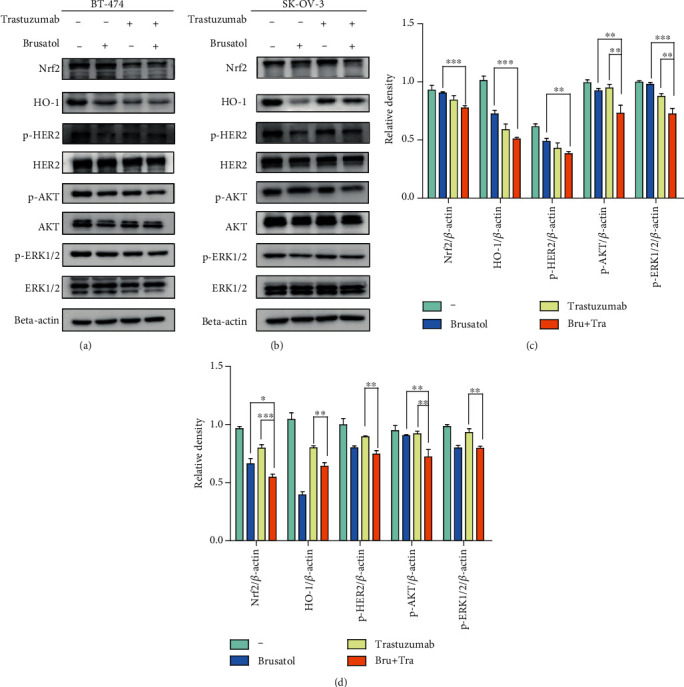
Trastuzumab plus brusatol potently inhibited the activation of Nrf2/HO-1 and HER2-AKT/ERK1/2 pathways. (a, b) BT-474 and SK-OV-3 cells were treated with trastuzumab or brusatol alone, or their combination for 24 hours. The changes in Nrf2/HO-1 and HER2-AKT/ERK1/2 signaling pathways were monitored by western blotting. (c, d) Densitometric analysis was performed on the western blotting. The levels of Nrf2, HO-1, p-HER2, p-AKT, and p-ERK1/2 were quantified by using the software Image J. The data are expressed as the mean ± SD of three independent experiments. ∗*p* < 0.05, ∗∗*p* < 0.01, ∗∗∗*p* < 0.001.

To explain the potential mechanism underlying the synergistical effect, we examined the inhibitory effects of trastuzumab, brusatol, or trastuzumab plus brusatol on HER2-AKT/ERK1/2 and Nrf2/HO-1 signaling pathway in BT-474 and SK-OV-3 cell lines. Results showing that trastuzumab plus brusatol effectively downregulates phosphorylation-HER2, as well as phosphorylation-ERK1/2 and phospho-AKT. Meanwhile, Nrf2 and HO-1 level was regressed in the two HER2-positive cell lines upon treatment with the combination therapy (Figures 3(a)–3(d)). These results suggested that brusatol may enhance the antitumor effect of trastuzumab through inhibiting the Nrf2/HO-1 and HER2-AKT/ERK1/2 signaling pathway in HER2-positive cancer cells.

**Figure 4:**
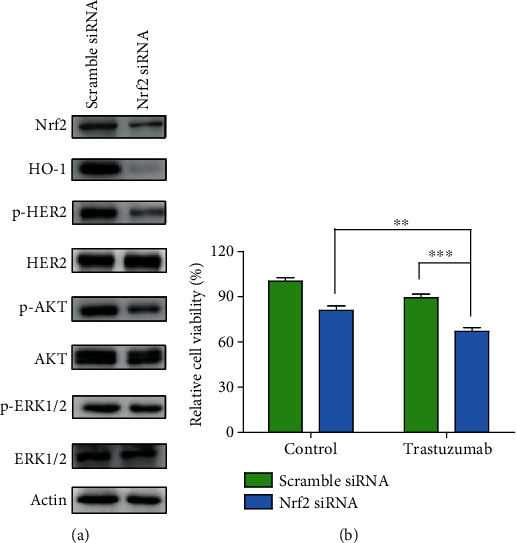
Nrf2 inhibition resulted in repression of HER2-AKT/ERK1/2 signaling and sensitizes SK-OV-3 cells to trastuzumab treatment. (a) The level of p-HER2, p-AKT, and p-ERK1/2 were determined after treatment with Nrf2-targeted siRNA or scramble siRNA for 36 h. (b) Cell viability was monitored after trastuzumab (50 *μ*g/mL) incubation for 24 h in scramble siRNA or Nrf2 siRNA-transfected cells. Cells were transfected with scramble siRNA or Nrf2 siRNA using Lipofectamine 2000 (Invitrogen) according to the supplier's instruction. Data show the mean ± SD (three independent experiments). ∗∗*p* < 0.01; ∗∗∗*p* < 0.001.

To further verify brusatol regressed HER2-AKT/ERK1/2 signaling by targeting Nrf2, we silenced Nrf2 expression using siRNA and examined the change of HER2-AKT/ERK1/2 signaling in SK-OV-3 cells. Immunoblot analysis showed that Nrf2 knockdown substantially decreased phospho-HER2 level, as well as phosphorylation-AKT and phosphor-ERK1/2 level in SK-OV-3 cells (Figure 4(a)). Subsequently, we tested if Nrf2 inhibition by Nrf2 knockdown will exert similar synergistic effects as brusatol on SK-OV-3 cells when cotreated with trastuzumab. In consistent with previous results, data showed that knockdown of Nrf2 also significantly enhanced the cytotoxic effect of trastuzumab in SK-OV-3 cells (Figure 4(b)). Overall, our results revealed that Nrf2 inhibition in combination with trastuzumab may be a promising strategy for treating HER2-positive cancers.

### 3.3. Trastuzumab Enhanced Brusatol-Induced Reactive Oxygen Species (ROS) Accumulation and Apoptosis in BT-474 and SK-OV-3 Cancer Cells

**Figure 5:**
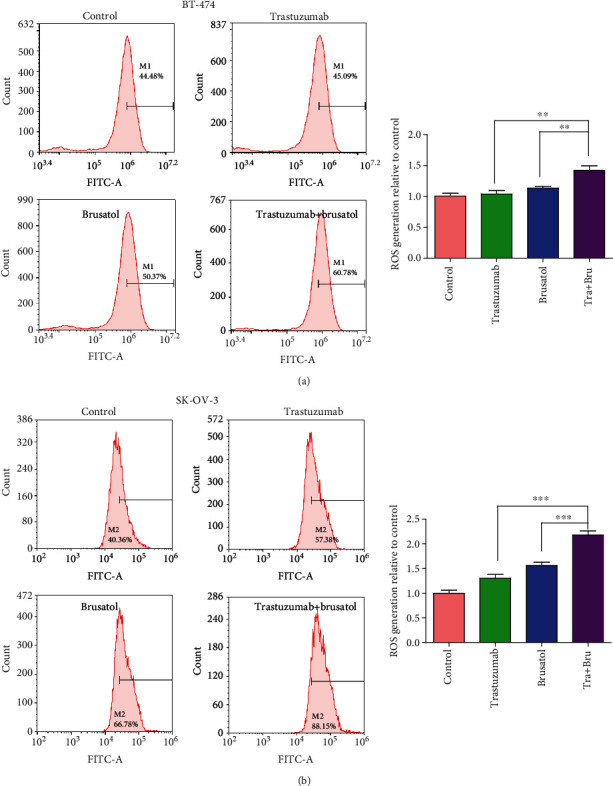
Trastuzumab in combination with brusatol effectively enhanced ROS accumulation in BT-474 and SK-OV-3 cells. (a) BT-474 were treated with control IgG (25 *μ*g/mL), trastuzumab (25 *μ*g/mL), brusatol (5 *μ*M), or trastzumab (25 *μ*g/mL) plus brusatol (5 *μ*M) for 24 h, and flow cytometry was used to analyze the level of ROS in cells after DCFH-DA was added to stain the cells. Bar graphic representations of the fluorescence intensity upon different treatments relative to control were shown. Data was presented as mean ± SD. (b) SK-OV-3 cells were treated with control IgG (25 *μ*g/mL), trastuzumab (25 *μ*g/mL), brusatol (5 *μ*M), or trastzumab (25 *μ*g/mL) plus brusatol (5 *μ*M), and flow cytometry was used to analyze the level of ROS in cells after DCFH-DA was added to stain the cells. Bar graphic representations of the fluorescence intensity upon different treatments relative to control were shown. Data was presented as mean ± SD.

Nrf2 suppression always caused elevation of ROS and subsequently resulted in apoptosis [34, 38, 39]. Next, we examined the level of ROS accumulation when BT-474 and SK-OV-3 cells were exposed to brusatol. Results showed that brusatol treatment alone led to a moderate ROS elevation in BT-474 and SK-OV-3 cells (Figures 5(a) and 5(b)). Surprisingly, the cotreatment of trastuzumab with brusatol resulted in a significantly greater increase on ROS accumulation than brusatol or trastuzumab treatment alone.

**Figure 6:**
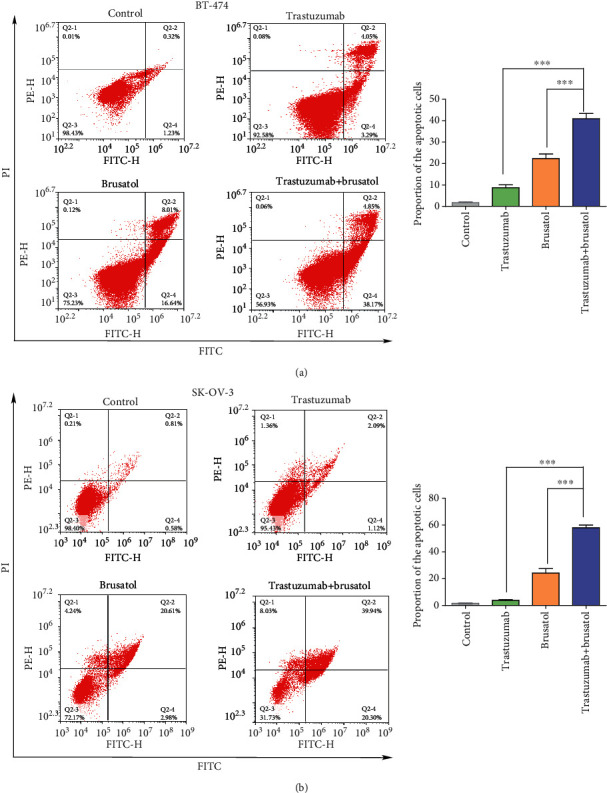
Trastuzumab in combination with brusatol potently induced apoptosis in BT-474 and SK-OV-3 cancer cells. (a) Induction of apoptosis of BT-474 cells after control IgG (25 *μ*g/mL), brusatol (5 *μ*M), trastuzumab (25 *μ*g/mL), or the combinatorial treatment for 48 h. Apoptosis ratios were measured by flow cytometry. Data was shown with mean ± SD of three independent experiments. ∗∗∗*p* < 0.001. (b) Induction of apoptosis of SK-OV-3 cells after control IgG (25 *μ*g/mL), brusatol (5 *μ*M), trastuzumab (25 *μ*g/mL), or the combinatorial treatment for 48 h. Apoptosis ratios were measured by flow cytometry. Data was shown with mean ± SD of three independent experiments. ∗∗∗*p* < 0.001.

As we know, ROS always takes an key role in exerting antitumor effects by activating apoptosis [40]. Thus, we examined if trastuzumab plus brusatol may potently induce apoptosis by utilizing FACS assay. Consistent with the results on ROS accumulation, data revealed that the combinatorial treatment more effectively enhanced apoptosis in both BT-474 and SK-OV-3 cell lines compared to treatment with either trastuzumab or brusatol treatment alone (Figures 6(a) and 6(b)). Taken together, these results suggested that the combination of trastuzumab with brusatol markedly promoted ROS accumulation and enhanced apoptosis in both BT-474 and SK-OV-3 cells, which may partly explain the superiority of combinatorial treatment.

### 3.4. Combination Therapy of Trastuzumab and Brusatol Is Superior to Single-Agent Treatment in HER2-Positive BT-474 and SK-OV-3 Cancer Cells *In Vivo*

**Figure 7:**
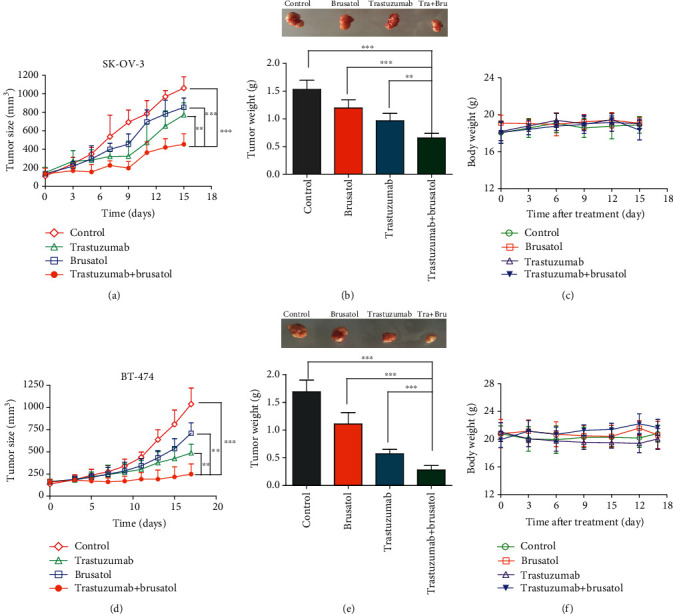
Trastuzumab caused tumor regression in BT-474 and SK-OV-3 xenografts in combination with brusatol. (a) Mean tumor volume of SK-OV-3 xenografts after injection with control IgG (15 mg/kg), trastuzumab (15 mg/kg), brusatol (2 mg/kg), or trastuzumab (15 mg/kg) plus brusatol (2 mg/kg). (b) On day 15 postfirst injection, xenograft tumors from each group were removed and tumor masses were weighed. (c) Effects of trastuzumab, brusatol or trastzuamb plus brusatol on tumor-bearing mice body weight were determined using SK-OV-3 tumor-bearing nude mice. Mice were weighed at regular intervals during the whole period to monitor unspecific toxicity. (d) Mean tumor volume of BT-474 xenografts after injection with control IgG (15 mg/kg), trastuzumab (15 mg/kg), brusatol (2 mg/kg), or trastuzumab (15 mg/kg) plus brusatol (2 mg/kg). (e) On day 17 postfirst injection, xenograft tumors from each group were removed and tumor masses were weighed. (f) Effects of trastuzumab, brusatol or trastzuamb plus brusatol on tumor-bearing mice body weight were determined using BT-474 tumor-bearing nude mice. Mice were weighed at regular intervals during the whole period to monitor unspecific toxicity. Data are shown asa mean ± SD. (*n* = 5 mice, each group); ∗*p* < 0.05; ∗∗*p* < 0.01; ∗∗∗*p* < 0.001.

Next, we examined the therapeutic efficacy of trastuzumab in combination with brusatol for nude mice bearing established BT-474 and SK-OV-3 tumor xenografts. As is shown in Figures 7(a) and 7(b), the antitumor activity of trastuzumab plus brusatol was significantly greater than that of trastuzumab or brusatol injection alone in SK-OV-3 xenograft tumors. Moreover, the drug combination treatment has not caused significant body weight loss in SK-OV-3 tumor xenografts (Figure 7(c)). In addition, we also evaluated the antitumor activity and nonspecific toxicity of trastuzumab plus brusatol in BT-474 xenografts. Results showed trastuzumab in combination with brusatol effectively regressed tumor growth and has not caused marked weight loss (Figures 7(d)–7(f)). Besides these, hematoxylin and eosin (H&E) staining also showed that no marked liver toxicity was observed in both BT-474 and SK-OV-3 tumor-bearing mice upon treatment with trastuzumab plus brusatol (Figures S1 and S2). Overall, these results above revealed that the combinatorial therapy shows superior antitumor effects and appear to be well tolerated in HER2-positive tumor xenografts.

**Figure 8:**
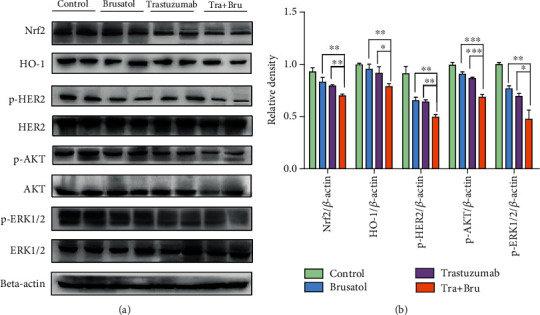
Combinatorial treatment with trastuzumab plus brusatol regressed Nrf2/HO-1 and HER2-AKT/ERK1/2 signaling pathways in SK-OV-3 tumor xenografts. (a) Tumors removed from SK-OV-3 xenografts upon treatment with control IgG (15 mg/kg), trastuzumab (15 mg/kg), brusatol (2 mg/kg), or trastuzumab (15 mg/kg) plus brusatol (2 mg/kg) were then subjected to Western blotting to detect the change of a series of proteins involved in Nrf2/HO-1 and HER2-AKT/ERK1/2 pathways. Two representative samples from each treatment group were shown here. (b) Quantification of Western blotting signal intensity analysis is expressed relative to the *β*-actin loading control by using Image J software. Data show the mean ± SD (three independent experiments); ∗*p* < 0.05; ∗∗*p* < 0.01; ∗∗∗*p* < 0.001.

To further explore the mechanism underlying the superior effects of trastuzumab in combination with brusatol *in vivo*, we removed tumors from drug-treated animals and evaluated the expression of key molecules involved in multiple signaling pathways utilizing western blot assay. Results in Figure 8 showed that the combinatorial treatment effectively suppressed the expression of Nrf2 and HO-1 involved in Nrf2/HO-1 pathway *in vivo*. Moreover, data also revealed that phosphorylation levels of HER2, AKT, and ERK1/2 were also potently inhibited in the SK-OV-3 tumor xenografts, which were consistent with the results *in vitro*. Consequently, these results above suggested that these patients with HER2-positive cancers may benefit from a combinatorial treatment with trastuzumab plus brusatol.

## 4. Discussion

In recent years, Nrf2 has been deemed as an important and promising target in cancer therapy and many efforts have been made to seek therapeutic strategies directed to block the Nrf2 antioxidant pathway [20, 41]. Brusatol, as a unique Nrf2 inhibitor, has recently been shown to regress tumor burden through inhibiting Nrf2 signaling in several tumor models [31, 32, 42]. More importantly, brusatol exhibits the potential as an adjuvant drug to enhance the efficacy of chemotherapeutic such as gemcitabine or cisplatin in pancreatic cancer or lung cancer [31, 34]. In the study, we firstly found that brusatol was a potent antitumor compound against HER2-positive cancer cells. Notably, the inhibitory effect of brusatol on HER2-AKT/ERK1/2 signaling pathway in HER2-positive cancer cells was identified as a new mechanism, suggesting a therapeutic advantage for the use of brusatol in cancer therapy.

Although clinical benefit has been verified with trastuzumab, many patients with HER2-positive cancers do not respond to trastuzumab or developed resistance in a period of trastuzumab therapy. It has been a clinical obstacle concerning with HER2-targeted treatment for a long time. Therefore, more effective targeted-therapy strategies for HER2-positive cancers are necessary to overcome the limitation of trastuzumab in efficacy. Harris et al. found that single-agent trastuzumab results in a limited treatment benefit in ovarian cancers; however, a combination therapy including both chemotherapy drugs and HER2-targeted antibody provides a far better response [43]. Many studies have also indicated that new combinatorial regimens of trastuzumab with other antibodies or small-molecule inhibitors may contribute to enhance the efficacy of trastuzumab and overcome trastuzumab resistance [9, 44, 45]. Recently, Gambardella et al. showed that Nrf2 activation through RPS6 is related to resistance to anti-HER2 drugs in HER2-amplified gastric cancer models [46]. And our previous publication also indicated that nimotuzumab in combination with trastuzumab exerts synergistic effects on HER2-positive breast cancers by repressing Nrf2 signaling pathway and induced ROS generation [2]. Collectively, these evidences suggest that Nrf2 may be a key target in treating HER2-positive cancers. Previously, many studies have indicated that brusatol could be developed as a relatively safe and effective anticancer adjuvant drug by targeting Nrf2 [47–50]. In the present study, we for the first time found that brusatol synergistically enhanced the antitumor effects of trastuzumab in HER2-positive BT-474 and SK-OV-3 cancer cells *in vitro* and *in vivo*. And we also observed that trastuzumab enhanced brusatol-induced ROS accumulation and promoted apoptosis in these cells, which further explained the synergistic effects. However, we have not observed similar synergistic effects in SK-BR-3 cancer cells. As we found that there was no significant difference in HER2 and Nrf2 expression level among the three cancer cell lines in our preliminary experiment (data not shown), we speculated that Nrf2 signaling may be not a key signaling in regulating the growth of SK-BR-3 cancer cells, which may partly explain the different effects in the three HER2-positive cancer cells. Additionally, the effects of trastuzumab plus brusatol on trastuzumab-resistant patients warrants further investigation. Therefore, brusatol could be used as a potent adjuvant drug for enhancing the efficacy of HER2-targeted therapeutics in treating HER2-positive cancers.

Recently, some researchers have indicated that coregulatory roles of HER2/HER3, Nrf2, and ROS may exist in several types of cancers including breast cancers and ovarian cancer [26, 51, 52]. In our study, we observed that Nrf2 antioxidant signaling as well as HER2-AKT/ERK1/2 signaling pathway was inhibited upon treatment with combinatorial treatment in HER2-positive BT-474 and SK-OV-3 cells. And knockdown of Nrf2 resulted in a repression of HER2-AKT/ERK1/2 signaling pathway in SK-OV-3 cells. The results suggested that dual inhibition of Nrf2/HO-1 and HER2-AKT/ERK1/2 signaling pathways is needed for the optimal antitumor effect of trastuzumab. Further study will be aimed to explore the exact regulatory mechanism of HER2-AKT/ERK1/2 pathway associated with Nrf2 antioxidant response pathway in HER2-positive cancer cells, which will greatly contribute to develop new therapeutics and therapy strategy against HER2-positive cancers.

In summary, our findings highlight the important role of Nrf2 inhibition against HER2-positive tumors in cancer therapy; especially, brusatol as a novel Nrf2 inhibitor was capable of sensitizing HER2-positive cancer cells to trastuzumab *in vitro* and *in vivo*, suggesting a great potential of brusatol as an adjuvant drug in combination with trastuzumab in treating HER2-positive cancers.

## Data Availability

The data used to support the findings of this study are available from the corresponding author upon request.

## Supplementary Materials

Supplementary Materials Figure S1: histological examination was conducted in BT-474 tumor-bearing mice treated with trastuzumab, brusatol, or trastuzumab plus brusatol. Representative images (magnification, ×200) of livers from nude mice (*n* = 5) after injected with trastuzumab, brusatol, or trastuzumab plus brusatol were obtained by staining with hematoxylin and eosin. Scale bars, 100 *μ*m. Figure S2: histological examination was conducted in SK-OV-3 tumor-bearing mice treated with trastuzumab, brusatol or trastuzumab plus brusatol. The same experiment condition with BT-474 tumor-bearing mice was utilized.
