# Supplementary figures and images for: Nrf2 Inhibitor, Brusatol in Combination with Trastuzumab Exerts Synergistic Antitumor Activity in HER2-Positive Cancers by Inhibiting Nrf2/HO-1 and HER2-AKT/ERK1/2 Pathways
Source: Oxid Med Cell Longev. 2020 Jul 19;2020:9867595. doi: 10.1155/2020/9867595 (PMC7387975; doi:10.1155/2020/9867595)

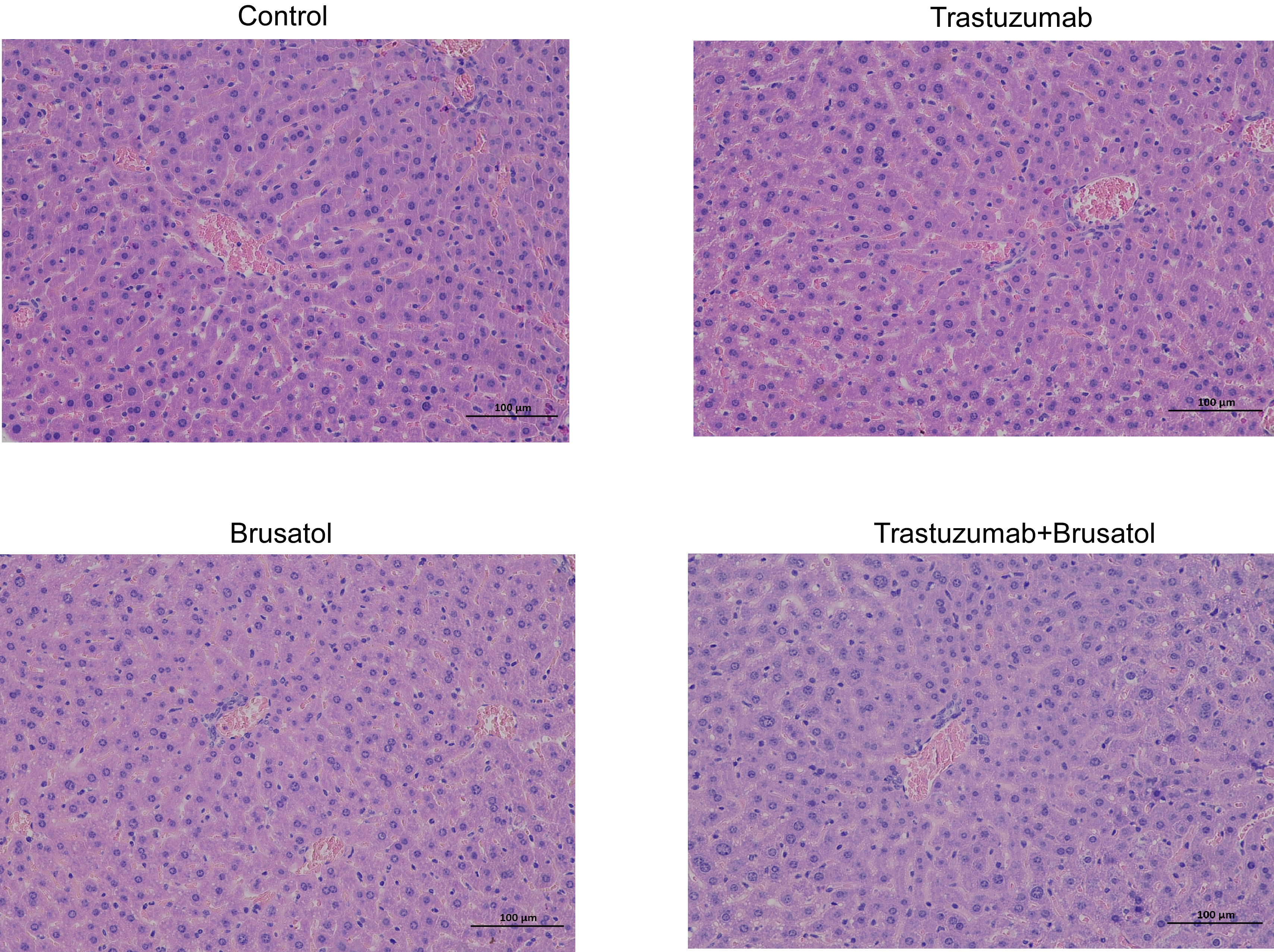

Supplement: Supplementary Materials — Figure S1: histological examination was conducted in BT-474 tumor-bearing mice treated with trastuzumab, brusatol, or trastuzumab plus brusatol. Representative images (magnification, ×200) of livers from nude mice (n = 5) after injected with trastuzumab, brusatol, or trastuzumab plus brusatol were obtained by staining with hematoxylin and eosin. Scale bars, 100 μm. Figure S2: histological examination was conducted in SK-OV-3 tumor-bearing mice treated with trastuzumab, brusatol or trastuzumab plus brusatol. The same experiment condition with BT-474 tumor-bearing mice was utilized. [file 9867595.f1.zip › Figure S1.tif]

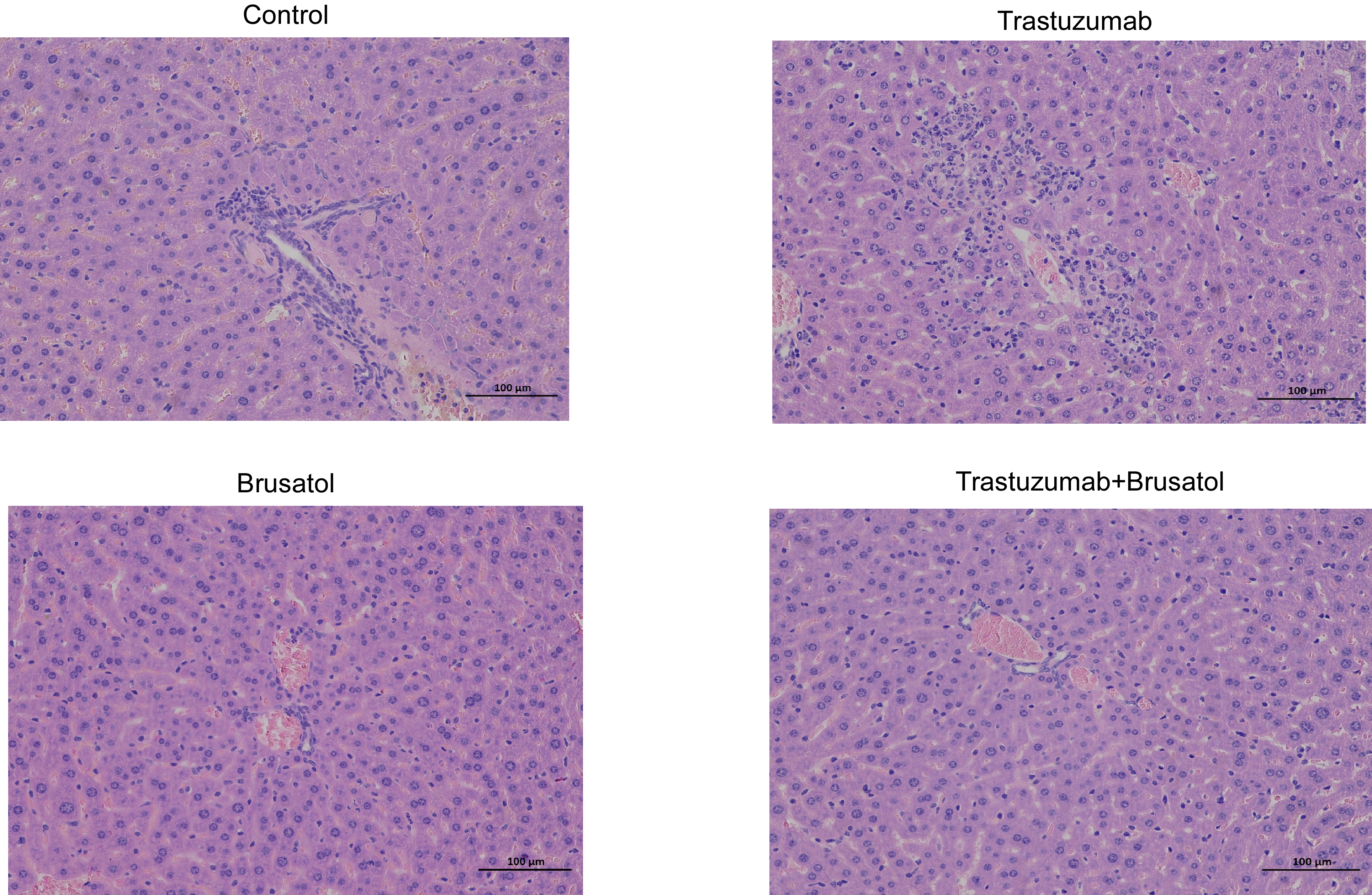

Supplement: Supplementary Materials — Figure S1: histological examination was conducted in BT-474 tumor-bearing mice treated with trastuzumab, brusatol, or trastuzumab plus brusatol. Representative images (magnification, ×200) of livers from nude mice (n = 5) after injected with trastuzumab, brusatol, or trastuzumab plus brusatol were obtained by staining with hematoxylin and eosin. Scale bars, 100 μm. Figure S2: histological examination was conducted in SK-OV-3 tumor-bearing mice treated with trastuzumab, brusatol or trastuzumab plus brusatol. The same experiment condition with BT-474 tumor-bearing mice was utilized. [file 9867595.f1.zip › Figure S2.tif]
